# An in silico modeling approach to understanding the dynamics of the post-burn immune response

**DOI:** 10.3389/fimmu.2024.1303776

**Published:** 2024-01-29

**Authors:** H. Ibrahim Korkmaz, Vivek M. Sheraton, Roland V. Bumbuc, Meifang Li, Anouk Pijpe, Patrick P. G. Mulder, Bouke K. H. L. Boekema, Evelien de Jong, Stephan G. F. Papendorp, Ruud Brands, Esther Middelkoop, Peter M. A. Sloot, Paul P. M. van Zuijlen

**Affiliations:** ^1^ Department of Plastic, Reconstructive and Hand Surgery, Amsterdam Movement Sciences (AMS) Institute, Amsterdam University Medical Center (UMC), Location VUmc, Amsterdam, Netherlands; ^2^ Department of Molecular Cell Biology and Immunology, Amsterdam Infection and Immunity (AII) Institute, Amsterdam University Medical Center (UMC), Location VUmc, Amsterdam, Netherlands; ^3^ Burn Center and Department of Plastic and Reconstructive Surgery, Red Cross Hospital, Beverwijk, Netherlands; ^4^ Preclinical Research, Association of Dutch Burn Centres (ADBC), Beverwijk, Netherlands; ^5^ Computational Science Lab, Informatics Institute, University of Amsterdam, UvA - LAB42, Amsterdam, Netherlands; ^6^ Center for Experimental and Molecular Medicine (CEMM), Amsterdam University Medical Center (UMC), Amsterdam, Netherlands; ^7^ Laboratory for Experimental Oncology and Radiobiology, ONCODE, Amsterdam University Medical Center (UMC), Location AMC, Amsterdam, Netherlands; ^8^ Laboratory of Medical Immunology, Department of Laboratory Medicine, Radboud University Medical Center, Nijmegen, Netherlands; ^9^ Department of Intensive Care, Red Cross Hospital, Beverwijk, Netherlands; ^10^ Complexity Institute, Nanyang Technological University, Singapore, Singapore; ^11^ Alloksys Life Sciences BV, Wageningen, Netherlands; ^12^ Paediatric Surgical Centre, Emma Children’s Hospital, Amsterdam University Medical Center (UMC), Location AMC, Amsterdam, Netherlands

**Keywords:** burns, wound healing, inflammation, immune response, computational modeling

## Abstract

**Introduction:**

Burns are characterized by a massive and prolonged acute inflammation, which persists for up to months after the initial trauma. Due to the complexity of the inflammatory process, Predicting the dynamics of wound healing process can be challenging for burn injuries. The aim of this study was to develop simulation models for the post-burn immune response based on (pre)clinical data.

**Methods:**

The simulation domain was separated into blood and tissue compartments. Each of these compartments contained solutes and cell agents. Solutes comprise pro-inflammatory cytokines, anti-inflammatory cytokines and inflammation triggering factors. The solutes diffuse around the domain based on their concentration profiles. The cells include mast cells, neutrophils, and macrophages, and were modeled as independent agents. The cells are motile and exhibit chemotaxis based on concentrations gradients of the solutes. In addition, the cells secrete various solutes that in turn alter the dynamics and responses of the burn wound system.

**Results:**

We developed an Glazier-Graner-Hogeweg method-based model (GGH) to capture the complexities associated with the dynamics of inflammation after burn injuries, including changes in cell counts and cytokine levels. Through simulations from day 0 – 4 post-burn, we successfully identified key factors influencing the acute inflammatory response, i.e., the initial number of endothelial cells, the chemotaxis threshold, and the level of chemoattractants.

**Conclusion:**

Our findings highlight the pivotal role of the initial endothelial cell count as a key parameter for intensity of inflammation and progression of acute inflammation, 0 – 4 days post-burn.

## 1 Introduction

Burns are a significant problem worldwide, having a high mortality and morbidity rate. Especially severe burns generally induce a massive long-term inflammatory response both systemically and locally (1–5).

Even though the inflammatory response is initially indispensable during wound healing, a massive persistent inflammatory response not only negatively affects the wound healing but may also result in multiple organ dysfunction (5). Despite significant progress in unravelling cellular and molecular processes involved in burn wound healing and the post-burn inflammatory response, the pathophysiology of burns and how, in this regard, burns differ from other non-burn wounds is far from fully understood.

The immune system is a key factor in wound healing and tissue regeneration. In the case of severe burns, the post-burn immune response is complex and involves intricate interactions between various cellular and molecular components (6, 7). It is essential to understand the dynamics of the immune response in burn wounds for the development of effective therapeutic interventions to improve patient outcomes (8–10). At the macroscopic level (i.e. patient level), the long-term inflammation that follows burn wounds often leads to deformities that affect the quality of life from burn patients. In severe cases, delayed wound closure (11), abnormal scarring (12), increased fibrosis (13), increased vascular proliferation (14) and excessive extracellular matrix deposition (12) can be caused by intense local and systemic inflammatory reactions.

**Figure 1:**
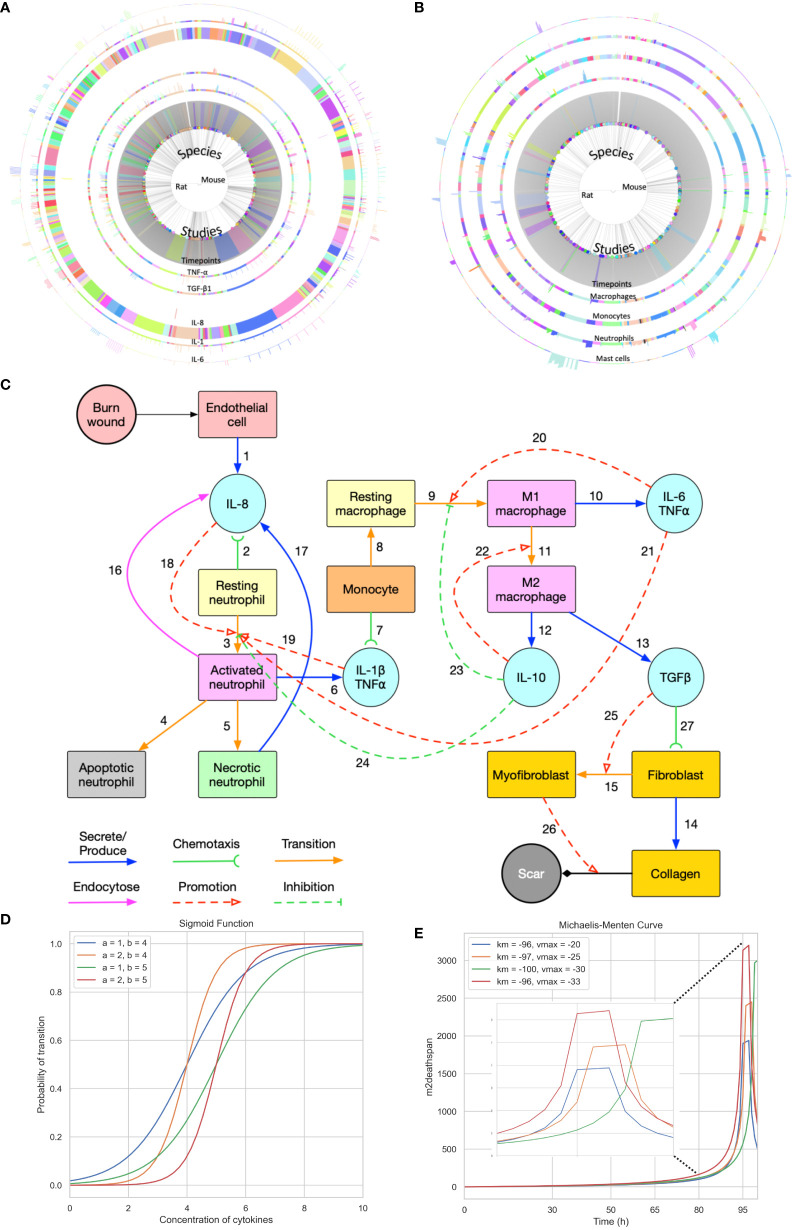
Summary of the data used for validation, conceptual model of the modelling approach, and sigmoid function and Michaelis-Menten function with different parameters. **(A)** Cell data relating different species to their respective cell characteristics pre-burn and after burn essay over time series by different studies. **(B)** Cytokine data relating different species to their respective cell characteristics pre-burn and after burn essay over time series by different studies. **(A, B)** Each arc represents different histogram data related to the main characteristic in study, note that this data has been obtained experimentally *in vivo.* **(C)** Conceptual model of the modelling approach. Each stock contains the variable in question and each link represents its relationship between variables (Created with BioRender.com). **(D, E)** Sigmoid function and Michaelis-Menten function with different parameters, respectively. Each value was randomized to show how different inflection points output different curves.

From a cell biology point of view, the number of inflammatory cells such as neutrophils, monocytes, macrophages, and level of pro-inflammatory cytokines are important at the acute inflammation stage of post-burn wound healing (4–6, 15, 16). During the acute inflammatory phase, between 24 and 72 hours, damage-associated molecular patterns (DAMP) and pathogen-associated molecular patterns (PAMP) trigger the immune response. This is accompanied by the release of interleukin (IL)-8, IL-6, tumor necrosis factor (TNF)α, IL-1β and IL-10, this process is summarized in **Figure 1C**. After 72 hours, macrophages are attracted to the wound site and differentiate into pro-inflammatory (M1) or “pro-healing” (M2) phenotypes. Fibroblasts migrate to the wound site, where angiogenesis takes place, with the help of endothelial cells. The processes that follow over the next days include reepithelization, revascularization, peripheral nerve repair, collagen fiber organization, reducing the number of macrophages and fibroblasts. Both decreased inflammation (i.e. normalization of the acute inflammatory mediators such as IL-6 and IL-8) and decreased angiogenesis will lead to limited scar formation (17). Endothelial cells play a crucial role in balancing the level of angiogenesis (18), helping to facilitate the inflammatory response and wound healing (19). After a burn injury, endothelial cells are among the first responders at the site of tissue damage. Among their main functions, they initiate the inflammatory response during the inflammation phase by expressing adhesion molecules and chemokines (20), facilitating the recruitment of immune cells such as neutrophils and monocytes to the wound area (21). Since endothelial cells are crucial during the acute inflammatory phase post-burn, we hypothesized that the endothelial cell count is one of the crucial parameters in burn wound healing and the acute immune response as shown in **Figure 1C**. Another key parameter is the time frame in wound healing.

Until now, both experimental and clinical approaches have been used to gain insight into the post-burn wound healing and specifically the immune response. However, due to the complexity of the involved processes, cellular and molecular pathways, these approaches have limitations such as the ability to sufficiently understand the underlying mechanisms to predict the system’s behavior. In some cases, it may not be possible to extract details on a finer-scale, such as spatial concentrations of cytokines at microscale level through animal experiments. Moreover, there are discrepancies in translating knowledge on cellular/molecular level for development of effective therapies for burn patients. To bridge this knowledge gap, computational modeling approaches have been developed as powerful tools to better understand complex biological processes (22–24). Recently, black box approaches such as machine learning and neural networks have been developed in context of burn wound healing process (25). Most of black box models in literature focus on wound healing prediction via image analysis and often overlook the underlying mechanisms driving the healing process. Burn wound healing, in general, wound healing, could benefit vastly from mechanistic computational models focusing on different aspects of the healing process. Turley et al. provide a comprehensive overview of how mathematical model techniques could play a role in exploring various aspects of wound healing including inflammation, closure and angiogenesis (26). Nagaraja et al. developed mechanistic models to understand the prolonged effects of inflammation (chronic inflammation) on the healing process and to test possible intervention strategies computationally (27, 28). These models were further expanded to identify biomarkers of pathological scarring in skin tissues (29). Though the above models are not specific to burn injuries, they showcase the usefulness of mathematical models in exploration and mechanism driven predictions of wound healing. In this study, we introduce an *in silico* mechanistic modeling approach to investigate the dynamics of the post-burn immune response. Agent-based modeling (ABM) techniques, specifically the Glazier-Graner-Hogeweg (GGH) model (30), is used to simulate the behavior of inflammatory agents and the dynamics of entities involved in burn wound inflammation (31, 32). By combining relevant experimental knowledge, data from existing experimental animal burn models, and mathematical principles (33, 34), our approach provides a comprehensive framework for studying the intricate dynamics of the (acute) immune response in burn wounds. Further, in this study, we hypothesize that endothelial cells act as one of the key players in determining the extent and duration of post-burn inflammation response. To verify this hypothesis, we use the developed spatio-temporal model to numerically simulate, analyze and qualitatively validate post-born immune responses during the acute inflammation phase.

## 2 Methods

### 2.1 Data description, analysis, and processing

The dataset provided by Mulder et al. (2), encompasses a collection of cytokine levels and immune cell counts of 14 different cell types from 247 studies involving rats and mice (**Figures 1A, B**). These measurements were taken across various animal characteristics (age, sex, species), burn wound characteristics (% total burn surface area, thickness and depth), sample source (in the skin), repeated samples, baseline comparison used, relative burn wound area, wound status (contraction, re-epithelialization, or overall burn area), anesthetic type used, and animal outcomes (healthy or not). Additionally, cytokine data specifically includes details on the analysis methods and the different cytokines measured, while cell count data specifies the method of inference and measurement. Both types of data are longitudinal follow-ups for some of the animals, which means that cell counts, and concentration of cytokines are accompanied by timeseries.

Notably, the dataset exhibits significant gaps, missing cytokine concentration or cell count reports for a notable amount of time frames, challenging the preprocess of data from a mechanistic standpoint. To address this issue, certain assumptions had to be made to enable specific validation points within the analysis. **Table 1** is the result of the raw data analysis on cell count change over time without any assumptions. These assumptions were made while considering the overall integrity and scientific validity of the data. Going forward, the model parameters were adjusted accordingly to fit the values provided by the data.

**Table 1:** Cell count transitions 0 – 4 days post-burn.

Cell type	0h	T	24h	T	48h	T	72h	T	96h	T
Resting Neutrophils (RN)	–	*↗*	–	*↘*	–	*↓*	–	–	–	–
Monocytes (M)	–	–	–	*↑*	–	*↘*	–	–	–	–
Fibroblasts (F)	–	–	–	–	–	–	–	*↑*	–	–
Activated neutrophils (AN)	–	*↑*	–	*↘*	–	*↓*	–	–	–	–
Necrotic neutrophils (NN)	–	–	–	–	–	–	–	–	–	–
Resting Macrophages (RM)	–	–	–	–	–	–	–	–	–	–
Macrophages type I (M1)	–	–	–	–	–	*↑*	–	*↘*	–	–
Macrophages type II (M2)	–	–	–	–	–	–	–	*↗*	*↗*	*↑*
Myofibroblasts (My)	–	–	–	–	–	–	–	–	–	*↗*

The symbols - , ↗, ↘, ↑, and ↓ indicate unknowns, growth, decline, increase, and decrease respectively, offering an intricate portrayal of cellular dynamics. T represents the transition between timepoints. Assumptions made based on data supplied by Mulder et al. (2).

### 2.2 Modeling approach

ABM is a computational modeling technique that enables the simulation of complex systems by representing individual agents and their interactions within a defined environment. ABM has gained increasing popularity in immunology (35, 36) because of its ability to capture the spatial heterogeneity and emergent behaviors observed in biological systems.

The GGH model, also known as the Cellular Potts Model (CPM), is a versatile computational method that enables the representation of non-uniform cell shapes as agents in multi-cell systems. It is a widely adopted ABM framework that has been successfully applied to simulate various biological phenomena, including cell migration (37), chemotaxis (38), and cell-cell interactions (39). This model is particularly suitable for studying the complex dynamics of the immune response, as it allows the representation of multiple cell types, the diffusion of soluble factors and the integration of experimental data extracted from existing *in vivo* animal burn models (rats and mice) (2) into the simulation framework (**Figures 1A, B**). With this integration, we expect to be able to find a good estimate for the parameters used to simulate the post-burn effects on cytokines, cells, and two-dimensional development of the wound. Our condensed two-dimensional simulation of the wound development implements the free energy mechanisms previously proposed using CompuCell3D (40, 41), an open-source GGH implementation.

#### 2.2.1 Simulation dynamics

The burn wound site is created in a 2D 5*5 cm square domain that includes two main components: the central 4*4 cm square represents the tissue component whereas the surrounding area of the central square is treated as the blood component (concept shown in **Figure 1C**). Because inflammation after burns intensifies during the first three to four days, the whole simulation will perform 1,000,001 Monte Carlo steps (MCS) with each 10,000 MCS representing one hour so that one simulation is treated as 100 hours in real time. The cells used in the simulation environment follow the rules of the conceptual model of **Figure 1C**, explained by following the links. Resting neutrophils and monocytes circulate commonly in the blood when there is no injury. If a burn occurs, the endothelial cells of the tissue component will secrete IL-8 [Link 1] to trigger the inflammatory response. Resting neutrophils are recruited from blood to tissue by chemotaxis to IL-8 [Link 2] and are activated by IL-8 [Links 3 and 18] when entering the tissue layer. Subsequently, activated neutrophils secrete pro-inflammatory cytokines IL-1β and TNF-α [Link 6] and these pro-inflammatory cytokines contribute to neutrophil activation [Link 19] and enhance the inflammatory response. Activated neutrophils can also endocytose IL-8 [Link 16] to neutralize inflammation to some extent. Meanwhile, activated neutrophils are short-lived, so they will enter apoptosis [Link 4] during inflammation. If inflammation persists, some activated neutrophils will turn into necrotic neutrophils [Link 5], triggering the release of additional IL-8 in the tissue [Link 17].

Attracted from blood to tissue, monocytes migrate toward IL−1β and TNF-α, secreted by activated neutrophils [Link 7], and then turn into resting macrophages, also called resting monocytes, in the tissue compartment in the presence of IL-6 [Link 8]. The resting macrophages are then transformed into activated macrophages [Link 9], commonly known as M1 macrophages, promoted by IL-6 and TNF-α. The macrophages of the M1 phenotype can secrete pro-inflammatory cytokines IL-6 and TNF-α [Link 10], which promote the activation of neutrophils and macrophages [Links 20 and 21]. M1 macrophages transition to M2 macrophages [Link 11] is promoted by the anti-inflammatory cytokine IL-10. M2 macrophages secrete IL-10 [Link 12] and TGF-β1 [Link 13]. In the current conceptual model, we only consider activated TGF-β1 (no latent), and IL-10 inhibits neutrophil and macrophage activation [Links 23 and 24]. Fibroblasts migrate towards TGF-β1 [Link 27] and can differentiate into myofibroblasts in the presence of (activated) TGF-β1 [Links 15 and 25]. Fibroblasts are responsible for collagen production [Link 14] and myofibroblasts cooperate with fibroblasts to form a collagen matrix [Link 26].

#### 2.2.2 Cell distribution, movement, and transitions

In our modeling system, each cell is depicted as a point arranged on a lattice. To introduce randomness and fairness to the position of the cells, we allowed the exchange of lattice sites between adjacent cells at their boundaries. To ensure that each lattice site has an equal chance of undergoing potential exchange during the simulation, we evaluated each potential exchange in random order. This constitutes a Monte Carlo step (MCS) which serves as the unit of time in our simulation. We selected a square grid for the simulations for two main reasons, 1. easier coupling between the cell field and the cytokine field concentrations, solved using finite volume solver (FiPy) and 2. to reduce computational complexity and resource usage if a hexagonal grid were to be considered for the simulation.

All cell types involved in the simulation will move due to chemotaxis and their initial number on the lattice is fixed, except for endothelial cells (as shown in **Supplementary Table 1** estimated from experimental data (2)). The Chemotaxis plugin in CC3D calculates the change of energy associated with the movement of the pixels, with the parameter λ controlling the strength of chemotaxis. Λ is specified for each type of cell, representing the response speed to a certain chemical. The energy formula is modified with a saturation coefficient, a, to include saturation terms (Equation 1, where c (x_destination_) and c (x_source_) denote the chemical concentration in the source pixel and the destination pixel, respectively). The minimum chemical concentration for the initialization condition is defined by a, which is used as an indicator of potential resistance to chemotaxis. Resting neutrophils are attracted by IL-8, monocytes by IL-1β and TNF-α and fibroblasts by TGF-β1. We assume that a burn injury occurs on a non-delicate area of the human body and the skin surface is uniform. The starting point of the implemented model are the remaining endothelial cells after the burn injury. Therefore, we assume that these do not move, and their number is limited and does not change during the simulation. We also assume that no cell can move across the boundaries so they will disappear if they reach the boundaries. However, in order to keep the number of cells constant in the simulation, we implement periodic boundary conditions, where every time a cell reaches a boundary, the cell reappears on the other end of the boundary, hence keeping the count of the cells in the simulation constant.


(1) $$\text{Δ}E_{\text{chem }}=\lambda \left[\frac{c\left(x_{\text{source }}\right)}{a+c\left(x_{\text{source }}\right)}-\frac{c\left(x_{\text{destination }}\right)}{a+c\left(x_{\text{destination }}\right)}\right]$$

When it comes to cell transitions, all the details and effects of the cytokines involved can be found in **Table 2**. As outlined in the conceptual model, each cell is treated as an agent in the simulation, so there is a chance that this cell will transform into another cell type. To address this probability, we used the sigmoid function (42) (**Table 3**, also known as the logistic function; **Figure 1D**). This function can take any real value as input and output value in the range (0, 1), which is necessary to randomly assign a transition probability. The first point of inflection(*a*) and the second point of inflection(*b*) can be adjusted to the specific needs of the cell transition. Since the sigmoid function is used to simulate the probability of a single factor, all the cytokines of promotion and inhibition should be incorporated to calculate the final transition probability. Based on Equation 2, the final probability that the resting neutrophils become activated consisted of five sigmoid functions, each of which will give a result between 0 and 1 depending on the concentration of each kind of cytokine.

**Table 2:** Cell type transition and the influence of cytokines used in simulations.

Cell type (from)	Cell type (to)	Promotion cytokines	Inhibition cytokines
Resting neutrophil (*N_R_*)	Activated neutrophil (*N_A_*)	*IL −* 8, *IL −* 6, *IL−* 1*β*, *TNF_α_*	*IL −* 10
Monocyte (*M_o_*)	Resting macrophage (*M_R_*)	*IL −* 6	–
Resting macrophage (*M_R_*)	M1 macrophage (*M*_1_)	*TNF_α_*, *IL −* 6	*IL −* 10
M1 macrophage (*M*_1_)	M2 macrophage (*M*_2_)	*IL −* 10	–
Fibroblast (*fi*)	Myofibroblast (*myofi*)	*TGF_β1_*	–

**Table 3:** Function types and values used in the simulations. y[x-1] means the previously calculated value is used. Inflection points obtained experimentally.

Function	1^st^ Inflection point	2^nd^ Inflection point	Equation
Sigmoid	a = 1	b = 4	$y\;=\;\frac{1}{1+e^{-a\left(x-b\right)}}$
Michaelis-Menten	Km = -963211.7	Vmax = -17.79826	$y\;=\;\{\begin{array}{c} \left|\frac{Vmax\;-\;x}{Km\;\;+\;x}\right|,\;if\;Vmax\;x\;\neq Km\;+\;x\\ y\left[x-1\right],\;\;\;\;\;\;\;\;\;\;\;\;\;\;otherwise \end{array}$

The coefficients *w*_1_ *, w*_2_ *, w*_3_ *, w*_4_ must sum to 1 for the final probability to remain between 0 and 1, and *w*_5_ must not exceed 1. Similarly, *w*_7_ *, w*_8_ and *w*_9_ must not exceed 1 in Equation 4. Additionally, *w*_6_ *, w*_10_ *, w*_11_ must not exceed 1 since there is only one term in Equations 3, 6 respectively. A base probability of 0.1 is given in Equations 3, 5, 6 in the event that there is no IL-6 and IL-10 present, as macrophages M1 and M2 do not exist in that situation. The only remaining cell transition is for activated neutrophils to become necrotic. Since there is no simple method in the literature that can detect neutrophils undergoing necrosis (43), activated neutrophils are assumed to be necrotic with a random probability (e.g., 0.1) in simulation.

The concentration of cytokines in each grid will be determined by solving the solute diffusion equations, and the cell transition will be based on probability equations. The parameters for the sigmoid function can be found in **Table 3**. Additionally, the order of magnitude of the cytokine concentration is essential to guarantee the accuracy of the calculations. Multiple pre-simulations were performed to determine the order of magnitude of the cytokine concentrations, which can be seen in **Supplementary Table 2**.


(2) $$P_{\left(N_{R}-N_{A}\right)}=w_{1}{\text{σ}}_{IL-8}+w_{2}{\text{σ}}_{IL-1\text{β}}+w_{3}{\text{σ}}_{IL-6}+w_{4}{\text{σ}}_{TNF\text{α}}-w_{5}{\text{σ}}_{IL-10}$$

(3) $$P_{\left(M_{o}-M_{r}\right)}=P_{base}+w_{6}{\text{σ}}_{IL-6}$$

(4) $$P_{\left(M_{R}-M_{1}\right)}=P_{base}+w_{7}{\text{σ}}_{IL-6}+w_{8}{\text{σ}}_{TNF\text{α}}-w_{9}{\text{σ}}_{IL-10\;}$$

(5) $$P_{\left(M_{1}-M_{2}\right)}=P_{base}+w_{10}{\text{σ}}_{IL-10}$$

(6) $$P_{\left(fi-myofi\right)}=w_{11}{\text{σ}}_{TGF-\text{β}1}$$

Some cells, such as resting neutrophils and monocytes, have a predetermined lifespan that is determined by biology (see **Supplementary Table 1 in the Supplementary Material**). On the contrary, the lifespan of other cells, such as fibroblasts and macrophages, is more difficult to measure due to the variability between individuals. However, certain assumptions had to me made to calculate changes in cell count during the healing process. For example, if in timepoint A the solute concentration or cell number is increased, and in timepoint B, the solute concentration or cell number is decreased(assuming A<B), then between these two points, the solute concentration or cell count is decreasing. By examining the data presented in **Figures 1A, B**, we can establish these assumptions (**Table 1**) and fit a Michaelis-Menten curve (44) specifically for macrophages type 2, which are of great importance in the immune response (45). Type 2 macrophages live beyond day 4 of simulation, however these cells have a significant impact on the downstream of burn wound healing. Assuming these are immortal, does not replicate the behavior in **Table 1**.Therefore, we assume that their immortality is not constant and tested different parameters and functions that can make sure the dynamics are replicated. The parameters and functions used are shown in **Table 3**.

#### 2.2.3 Model assumptions

In this model we simulate the burn wound microenvironment after it has occurred, therefore at the initial point(t=0), we assume the start of the process of healing after burn injury. Owing to the two dimensional nature of the model, it does not include the depth or proliferation within the wound. To model accurately this wound patch of burn injury, we assume that the burn wound is located on a non-delicate area of the human skin, therefore the injury has not occurred on the face, genitals, or fingertips, for example. We consider a limited supply of cells in circulation and in the wound patch simulated (16cm^2^). This assumption could be replaced with experimentally or clinically observed vessel density metrics from the wound area in future studies. The exchange of cells and solute between the wound area and the blood supply is possible, and this is driven by the interactions described in **Figure 1C**. The blood supply is limited only by the amount of circulating solute concentration or cells around the wound patch, therefore it implies that more cells can still be recruited. We allow cells to die by either transitioning into necrotic or apoptotic(only in neutrophils) or disappearing from the wound when reaching certain volume. A comprehensive data availability on cell count from the burn wound tissue and blood sample would help side step the above assumption in future models. In the current model, whenever a cell disappears, a new one takes its place, keeping the cell count homogenous across simulations. The severity of the wound is dictated by the concentration of pro-inflammatory cytokines vs anti-inflammatory cytokines given a certain timespan. Finally, we take into consideration only the presence of Fibroblasts and Myofibroblasts, and not collagen or scar formation (**Figure 1C**), since we limit the model to the effects on the wound path post-burn injury.

#### 2.2.4 Differential equations

There are six main solute molecules described in the conceptual model. The diffusion of IL-8 (Equation 7), IL-1β (Equation 8), IL-6 (Equation 9), IL-10 (Equation 10), (Equation 11) and TGF-β1 (Equation 12) with respect to time. All equations contain one diffusion term (*D_cytokine_*), one or two secretion terms (*K_cytokine_*), and one decay term (*μ_cytokine_*). The values of diffusion coefficient, decay rate, secretion rate, and endocytosis rate (θ*_cytokine_*) are found in the literature and adapted to suit the same order of magnitude in the model. All these diffusion equations are solved within each MCS during the simulation. Fixed gradient boundary conditions (Neumann) are specified in the top, right, bottom and left corners of the whole lattice, meaning that the derivative at a boundary is zero or a constant. The cell number parameters in the equations, such as the number of endothelial cells (EC), necrotic neutrophils (N_N_), and activated neutrophils (N_A_), macrophages type 1 (M_1_), macrophages type 2 (M_2_) at time t, depend on the presence of the cells in the location where the cytokine is being expressed and the initial values for each can be found in **Supplementary Table 3**.


(7) $$\frac{\partial \phi_{IL-8}}{\partial t}=\;D_{IL-8}\nabla^{2}{\;}_{\phi IL-8}-\mu_{IL-8\phi IL-8}+K_{EC|IL-8}EC+\;K_{N{\;}_{N}|IL-8}N_{N}-\theta_{N_{A}|IL8}N_{A}$$

(8) $$\frac{\partial \phi_{IL-1\beta }}{\partial t}=\;D_{IL-1\beta }\nabla^{2}{\;}_{\phi IL-1\beta }-\mu_{IL-1\beta }\phi_{IL-1\beta }+K_{N_{A}|IL-1\beta }N_{A}$$

(9) $$\frac{\partial \phi_{IL-6}}{\partial t}=\;D_{IL-6}\nabla^{2}{\;}_{\phi IL-6}-\mu_{IL-6}\phi_{IL-6}+K_{M_{1}|IL-6}M_{1}$$

(10) $$\frac{\partial \phi_{IL-10}}{\partial t}=\;D_{IL-10}\nabla^{2}{\;}_{\phi IL-10}-\mu_{IL-10}\phi_{IL-10}+K_{M_{2}|IL-10}M_{1}$$

(11) $$\frac{\partial \phi_{TNF\alpha }}{\partial t}=\;D_{TNF\alpha }\nabla^{2}{\;}_{\phi TNF\alpha }-\mu_{TNF\alpha }\phi_{TNF\alpha }+K_{N_{A|TNF\alpha }}N_{A}+\;K_{M_{1}|TNF\alpha }M_{1}$$

(12) $$\frac{\partial \phi_{TGF\beta 1}}{\partial t}=\;D_{TGF\beta 1}\nabla^{2}{\;}_{\phi TGF\beta 1}-\mu_{TGF\beta 1}\phi_{TGF\beta 1}+K_{M_{2}|TGF\beta 1}M_{2}$$

### 2.3 Statistical analysis

We employed the Z-score from the *SciPy* package due to its key benefits (46). This approach offers scale independence, making data unit-independent and allowing comparison between variables with different units. The Z-score also provides interpretability, with each score indicating how many standard deviations (SD) a data point is from the mean. Moreover, the original distribution of the data is preserved. By using this method, we can guarantee that the data are on a common scale, which facilitates meaningful comparisons and statistical analyses. Mathematically, Z-scores (Z) are calculated by subtracting the mean (μ) from the data (X) and dividing by the standard deviation (σ), resulting in Equation 13.


(13) $$Z=\frac{X-\mu }{\sigma }$$

The suitability of this method with wide-ranged data made it an ideal choice for our research context.

### 2.4 Parameters

Firstly, a parameter scan (i.e. evaluation of possible parameters for the validation assumptions/data) was run thoroughly to verify the sensibility of the Saturation Coefficient and *λ* parameters. Followed by the experimental design, described in **Table 4**, for the variable in question, the endothelial cell count.

**Table 4:** Experimental design: Endothelial cell count in 8 groups.

Experiment	*S _1_*	*S _2_*	*S _3_*	*S _4_*	*S _5_*	*S _6_*	*S _7_*	*S _8_*
Endothelial count	10	100	500	1000	2000	3000	4000	5000
Lambda	2000							
Saturation coefficient	10^−11^							

### 2.5 Code, plugins, configurations and hardware used

The simulation code, configuration files, and additional results used in our study can be found in this respository .Programming was done in Python 3.10. All simulations were performed using Compucell3D software (version 4.4.1) on Macbook Pro 2019 with a 2,3 GHz 8-Core Intel Core i9 processor.

The chemical field used to simulate chemotaxis is incorporated with *Fipy* and the partial differential equations were solved by using the *LinearGMRESSolver()* function.

Additional modules in the CompuCell3D environments used in our simulations and their function are described in **Supplementary Table 1B**.

## 3 Results

### 3.1 Cell count transition 0 – 4 days post-burn

**Table 1** presents cell count transitions based on available data from the rat and mouse burn studies (2) in a time frame from 0 – 4 days post-burn. According to this, a lot of data points were unknown. Based on available data, resting neutrophils growth increased immediately (0h) after burn (increased Z-score), and resting neutrophil growth declined 24h post-burn (decreased Z-score). Monocytes number increased after 24h, and monocyte growth declined after 48h post-burn. Fibroblasts number increased after 72h post-burn. Activated neutrophils number increased immediately (0h) post-burn, activated neutrophils growth declined after 24h, and activated neutrophil number decreased after 48h post-burn. Macrophages type I (M1) number increased after 48h, M1 growth decreased after 72h post-burn. Macrophages type II (M2) growth increased after 72h up to 96h, and M2 number increased after 96h post-burn. Myofibroblasts growth increased after 96h post-burn.

### 3.2 Increasing endothelial cell count leads to opposite correlations between IL-8 and TGF-β1

**Figure 2:**
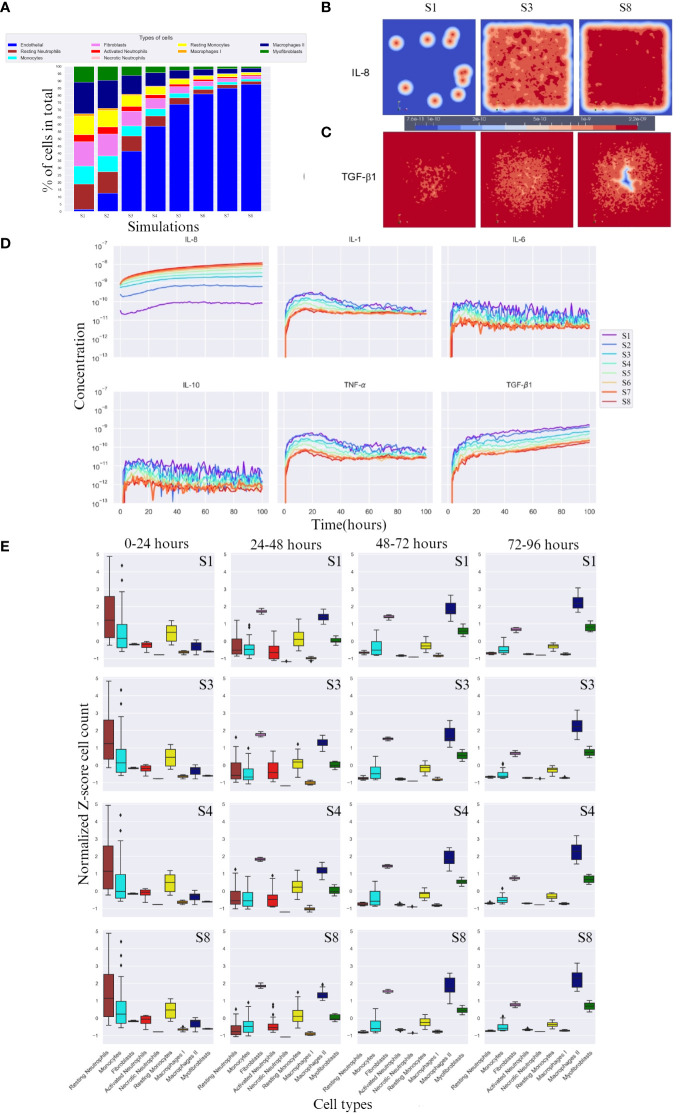
Overall cell type percentage per simulation, diffusion of IL-8 and TGF-β, and cytokine concentration and cell count over time. **(A)** Overall cell type percentage per S (simulation), note that endothelial cell count increases every S. **(B, C)** Graphic visualization of the diffusion of IL-8 and TGF-β1 in the 100th hour of simulation, respectively (scale for reference, values range within the same order of magnitude). **(D)** Cytokine concentration over time (in hours) per (S) simulation on a logarithmic scale (order of magnitude). **(E)** Breakdown of Z-score normalized cell count over four time frames between hours 0 and 96 for S1, S3, S4 and S8. Only most significant simulations are shown, i.e. where the change in cell count is more evident. For each box plot, the mean is plotted as a black line inside the box and the standard deviation located in each side of the box (at 95% CI), outliers shown as rhombus points. Note that the calculations for Z-score were done with respect to mean of each cell count/cytokine concentration. The score here indicates how many standard deviations an observation is from the mean.

After running the experimental design outlined in Section 2, we compared the cell percentages between simulations (**Figure 2A**). The percentage of necrotic neutrophils was very small compared to other cell types and almost indistinguishable. With increasing number of endothelial cell count, all cell types except necrotic neutrophils were decreased (due to the immortality of endothelial cells) in percentage (**Figure 2A**). We took snapshots at different time intervals for all cytokines, for different simulations (**Figures 2B, C**), with only the snapshots from the 100th hour (± 4 days post-burn) shown. With higher endothelial cell count, the diffusion of IL-8 towards the center, the site that corresponds with the burn wound, increased and the diffusion of TGF-β1 towards the centeE r decreased. This caused the relationship between IL-8 and TGF-β1 to become opposite in all simulations (**Figure 2D**). Specifically, the concentration of IL-8 increased from simulation 1 to 8 and TGF-β1 concentration decreased from simulation 1 to 8, compared in the same order of magnitude (taking highest concentration as comparison), over time.

### 3.3 Higher endothelial cell count results in higher IL-8 concentrations

IL-8, unlike TGF-β1, doubles in concentration with increasing endothelial cell count, reaching double the order of magnitude (**Figure 2D**). Evidently, for IL-8, a higher endothelial cell count leads to a faster equilibrium convergence around the 10*^−^* ^8^ order of magnitude of concentration, since S6 and S7 slowly converge to the concentration value achieved by S8.

### 3.4 Higher endothelial cell count resulted in increased cytokine response and cell activation

In the first 24 hours, a lower concentration of IL-8 led to delayed activated neutrophil recruitment, whereas a higher concentration led to a more rapid growth. TGF-β1 had the opposite reaction at higher concentrations. All simulations showed a similar average number of cells across simulations, suggesting an incoming decrease if the availability of cells is high, and an increase if the cells are needed in early stage (supplied by the blood compartment), evidenced by the high SD (**Figure 2E** 0-24 hours column).

After 24h, a higher endothelial cell count leads to increased cytokine response and cell activation for tissue repair. The diffusion of TGF-β1 increased, which was aided by the higher number of M1, and an increase in fibroblast number (**Figure 2E** 24-48 hours column). Consequently, activated neutrophils also decreased in cell count. Higher endothelial cell count also resulted in decreased cell count of monocytes.

Between 48 and 72 hours (**Figure 2E** 48-72 hours column), cytokine levels generally decreased as they reached equilibrium and began to influence cell responses. However, myofibroblast counts increased due to the increased presence of TGF-β1, transitioning from fibroblasts. M2 cell counts also increased.

In the last hours of the simulation, 72 – 96h (**Figure 2E** 72 – 96 hours column), endothelial cell count resulted in increased M2 cell count.

### 3.5 The physical and physiological reflection of endothelial cell count difference

**Figure 3:**
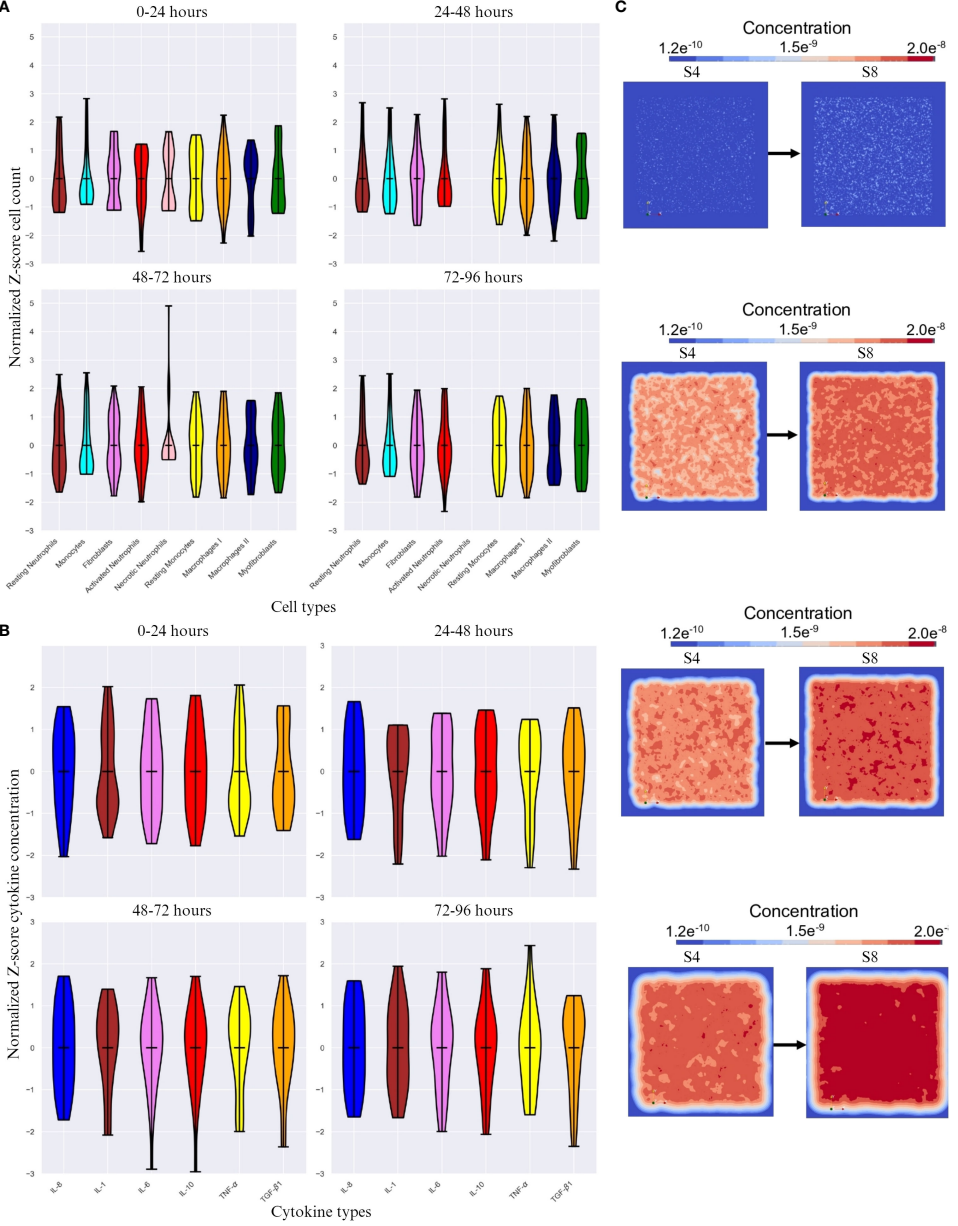
The difference in cell count and cytokine concentration over different time intervals. **(A)** Z-score normalized difference in cell count(y-axis) over different time intervals between simulation 8 (S8) and simulation 4 (S4 - control) for different cell types(x-axis). **(B)** Z-score normalized difference in cytokine concentration(y-axis) over different time frames. **(C)** Initial, 48 hour, 72 hour and 96 hour change in positional diffusion for TGF- β1 (from top to bottom) by comparing S4 (control, on the left) and S8 (on the right). Note that the calculations for Z-score were done with respect to mean of each cell count/cytokine concentration. The score here indicates how many standard deviations an observation is from the mean.

We chose a control simulation that showed similar dynamics to the indicated in **Table 1**. We then used the difference in cell count and cytokine concentration for analysis. **Figures 3A, B** show the Z-scored difference in cell counts and cytokine levels(respectively) between S8 and S4 (control) for different time frames. This provides a more detailed view of the effect of endothelial cell count.

#### 3.5.1 Significance of timing in the simulation environment

The cell count is expected to be highly variable, which is reflected in the high SD change for all cell types across time frames except necrotic neutrophils and myofibroblasts (**Figure 3A**), which either are not shown (24 – 48 and 72 – 96 timespans for necrotic neutrophils) or vary in Z-score very actively. With increasing endothelial cells, the cell types with the highest SD change are resting neutrophils, monocytes, resting monocytes, M1 and M2. The most significant difference was seen in the first 24 hours and the last 48 hours of the simulation, with M2 standing out as the most variable cell type across time intervals.

#### 3.5.2 Faster inflammation resolution is caused by the difference in endothelial cell count

A higher endothelial cell count led to faster resolution of inflammation, restricting the duration of the inflammatory phase (**Figures 3B, C**). Pro-inflammatory cytokines were the most active cytokines, whereas the other cytokines remained relatively stable, with minimal difference in TGF-β1 concentration. Despite this encounter, TGF-β1 as an anti-inflammatory cytokine, had most changes in SD during the last 48 hours (**Figure 3B**) showing inflammatory resolution spatially (**Figure 3C**).

## 4 Discussions

In previous studies, we and others have demonstrated that the (acute) immune response is disrupted, i.e., “over-active” and prolonged, in burn patients, which negatively affects the wound healing and can cause further significant complications systemically (1–5).

In this article, we proposed a comprehensive framework to study the intricate cellular and molecular dynamics of the immune response in burn wounds by using relevant biological knowledge, experimental data from existing animal burn models, and mathematical principles.

After burn, endothelial cells play a crucial role in the inflammation phase of the wound healing. Specifically, during the acute phase, endothelial cells facilitate the movement of circulating inflammatory cells into the tissue at the site of burn injury (47). Therefore, we hypothesized that the endothelial cell count is one of the crucial parameters in burn wound healing and the acute immune response. The conceptual model initiates post-burn at t = 0; meaning that this is the moment that the simulation starts after burn. Within the model, the cells can move (except endothelial cells), are randomly located (mimicking the real burn wound injury circumstances), secrete cytokines, and differentiate. The burn surface area is limited to 5 by 5 cm area.

With these assumptions we demonstrated that our model can simulate the temporal evolution of the cell counts of the various cell types involved in the wound healing process (e.g., endothelial cells, neutrophils, monocytes/macrophages), and the diffusion of cytokines was simulated over a period of 0 – 4 days post-burn. The resulting simulations were tested and validated by experimental data.

Based on available data from rat and mouse burn studies (2), we identified the cell count transitions 0 – 4 days post-burn. Although, data points at different time frames were missing (unknown); we had to make assumptions for the time points in between, based on known data points we were able to simulate the cell count transitions over time. This is a limitation of the model, but in reality, it is not experimentally feasible to get complete timeseries for cell count (and growth) for each cell type.

Our findings revealed a key role for endothelial cells count in the acute immune response, higher endothelial count led to increased cytokine response and cell activation. Since endothelial cells play a crucial role in the regulation of inflammation during the early stages of wound healing by facilitating the movement of inflammatory cells into the tissue, it can be expected that higher counts results in higher inflammatory reaction (e.g. increased cytokines and inflammatory cell activation) (48).

### 4.1 Simulation period 0-24 hours

In the first 24h of simulation, we found already a high number activated neutrophils, where lower concentration of IL-8 led to delayed activated neutrophil recruitment, and TGF-β1 had the opposite reaction (**Figures 2D, E**). These findings correlates well with findings in *in vivo* studies. Namely, in the first 24h post-burn, there is an enhanced neutrophil migration activity (49), IL-8 plays a causative role in the recruitment and activation of neutrophils (50), and TGF-β1 desensitize neutrophils to chemotaxic stimulation (48).

### 4.2 Simulation period 24 – 72 hours

24 – 48h of simulation we found that both resting and activated neutrophil numbers decreased, and monocytes number increased. After 48h, monocyte numbers decreased, activated neutrophil number decreased further, and macrophage type I number increased (**Figures 2D, E**). The increase of monocyte numbers after 24h are in line with what was found in studies with burn patients (3, 51, 52). From 48 – 72h after burn monocytes are expected to decrease since they differentiate into macrophages (53). However, neutrophils *in vivo* normally increase in count for a longer period after burn (50); especially burn wounds are characterized by a prolonged local acute inflammatory response of innate immune cells (3, 52). This can be explained by the fact that the supply of cells *in vivo* is higher than in the *in silico* setting, since the supply in the model indicates an exchange from the blood compartment to the tissue compartment, where we assume a limited number of cells in both compartments. This means that there is no “unlimited” supply of cells when e.g. neutrophils die by necrosis (apoptosis in not implemented yet into the model either).

### 4.3 Simulation period 72-96 hours

72 – 96h of simulation, macrophage type I number decreased, while macrophage type II number increased, and both fibroblasts and myofibroblasts numbers increased. Moreover, these phenomena were positively affected by TGF-β1 concentrations (**Figures 2D, E**). With higher endothelial cell count, the diffusion of IL-8 towards the center, the site that corresponds with the burn wound, increased and the diffusion of TGF-β1 towards the center decreased (**Figure 3**). These findings are in line with the proliferation/remodeling phase during wound repair and scar formation *in vivo*. The macrophage phenotype changes as the wound heals, progressing from the macrophage type I (pro-inflammatory) to the macrophage type II (anti-inflammatory) (54). Fibroblasts first proliferate, and thereafter differentiate into myofibroblasts, which contract and participate in healing by reducing the size of wound and secreting ECM proteins (55). Regarding this phase of wound repair, processes such as collagen production, wound closure, scar formation, contraction, are not implemented in the conceptual model yet. The current model simulates the cells that are mainly intervening in the (acute) inflammatory phase. And also for these cell types the supply of cells *in vivo* is higher than in the *in silico* setting.

The time frame in (burn) wound healing is another important parameter. In the current simulation this is limited by the 0 – 4 day time frame used to initially simulate the acute inflammatory phase of the post-burn immune response. Furthermore, in our model, we focused on the role of endothelial cells count. In addition to endothelial cells, e.g., thrombocytes play an important role in the inflammation phase of wound healing too (56). Thrombocytes provide high levels of platelet-derived growth factor (PDGF), which stimulates chemotaxis of monocytes/macrophages and proliferation of fibroblasts, both crucial during wound healing (56). However, thrombocytes are not included in the conceptual model yet, to simplify the modelling approach.

With regards to the limitations associated with the current model, most appear to arise from the biological proxies used in the model. For instance, some cell types are assumed to be immortal, this is practical considering the smaller duration of the total simulation period (4 days). However, in reality, some of these cells may die and release ITMs (Inflammation Triggering Moieties). These ITMs could further prolong the local inflammation duration at the wound sites. The extent of inflammation from such events could only be calculated from accurate experimental data of cell counts at different time periods. We assume that wound is in a non-sensitive area. In extensive burns, this may not be the case, thus new players not considered in the model could play a crucial role in the healing and wound closure processes. Finally, to lower computational complexity we have considered only innate immune system (cytokines and cells), the effect of adaptive system is not included, which may reduce the personalization aspect of the current model.

## 5 Conclusions

In this work, we provided a different perspective of the post-burn transition between cell types and the dynamics of the life span of cells that are difficult to measure experimentally. Although the availability of certain biological factors (data) was at some point limited, our model can simulate events that take place during the post-burn wound healing process can correlate with biological data with appreciable accuracy. The model simulation results are qualitatively verified against relevant studies in the literature. In a way, this is also one of the major limitation of the current model. A complete quantitative verification against experimental or clinical data is currently not possible either due to lack of complete longitudinal data or practical limitations in acquiring some datapoints (such as spatial cytokine concentrations) experimentally. The next step of model development is to investigate the relation with wound healing parameters, i.e., wound closure, re-epithelialization, and scar formation, and patient specific characteristics; and identify processes on long-term (after 4 days post-burn). This approach would provide a comprehensive quantitative validation against clinical data. Since the current model is developed as a conceptual dynamic model, which will be continuously fed by new (generated) data from micro- to macroscale, we expect it will be a continuous learning model for the cellular and molecular processes during the immune response after burn. We have focused on developing a spatio-temporal model to simulate the inflammatory phase of burn wound in this study. In future studies, we plan to computationally model and validate other aspects and timelines of burn wound healing including acute inflammation, collagen production and scar formation. Such individual yet standardized models would provide a platform to combine them later to simulate the whole process of wound healing. In addition, the open-source nature of the developed GGH models and simulation techniques, other research including omics studies and machine learning techniques could be integrated to the mechanistic approach with little computational effort. Eventually, this will enable to prediction of systems behaviors and clinical outcomes in the burn wound healing process.

## Data availability statement

The original contributions presented in the study are included in the article/**Supplementary Material**, requests to access these datasets should be directed to BB, bboekema@burns.nl.

## Ethics statement

Ethical approval was not required for the study involving animals in accordance with the local legislation and institutional requirements because we made use of published data from animal studies to validate our simulation models.

## Author contributions

HK: Conceptualization, Data curation, Formal analysis, Investigation, Methodology, Resources, Supervision, Writing – original draft, Writing – review & editing. VS: Conceptualization, Data curation, Formal analysis, Investigation, Methodology, Resources, Software, Supervision, Writing – original draft, Writing – review & editing. RVB: Data curation, Investigation, Methodology, Writing – original draft. ML: Data curation, Investigation, Methodology, Writing – review & editing. AP: Conceptualization, Writing – review & editing. PM: Conceptualization, Writing – review & editing. BB: Conceptualization, Writing – review & editing. EJ: Conceptualization, Writing – review & editing. SP: Conceptualization, Writing – review & editing. RB: Conceptualization, Writing – review & editing. EM: Conceptualization, Writing – review & editing. PS: Conceptualization, Supervision, Writing – review & editing. PZ: Conceptualization, Supervision, Writing – review & editing.
