# Supplementary material for: An in silico modeling approach to understanding the dynamics of the post-burn immune response
Source: Front Immunol. 2024 Jan 29;15:1303776. doi: 10.3389/fimmu.2024.1303776 (PMC10859697; doi:10.3389/fimmu.2024.1303776)
Supplement: Supplementary file 1 [file Table_1.docx]

**Supplementary**

**Table S1**: Summary of Terms and Description used in the model.

| **Equation Terms** | **Description** | **Value** |
| --- | --- | --- |

*Promotion or Inhibition saturation cytokine concentration (C) in g/mL*

| *ϕIL−*8 | *CIL−*8 | 2 x 10^-9^ |
| --- | --- | --- |
| *ϕIL−*1*β* | *CIL−*1*β* | 5 x 10^-9^ |
| *ϕIL−*6 | *CIL−*6 | 5 x 10^-9^ |
| *ϕIL−*10 | *CIL−*10 | 5 x 10^-9^ |
| *ϕTNF_α_* | *CTNF_α_* | 5 x 10^-9^ |
| *ϕTGF_β1_* | *CTGF_β1_* | 5 x 10^-9^ |

*Diffusion coefficient (DC) in*${cm}^{2}/s$

| *DIL−*8 | DC of IL-8 | 2*.*09 *×* 10*^−^*^6^ [31] |
| --- | --- | --- |
| *DIL−*1*β* | DC of *IL −* 1*β* | 3 *×* 10*^−^*^7^ [32] |
| *DIL−*6 | DC of IL-6 | 8*.*49 *×* 10*^−^*^8^ [33] |
| *DIL−*10 | DC of IL-10 | 1*.*45 *×* 10*^−^*^8^ [33] |
| *DTNF_α_* | DC of *TNF_α_* | 4*.*07 *×* 10*^−^*^9^ [33] |
| *DTGF_β1_* | DC of *TGF_β1_* | 2*.*6 *×* 10*^−^*^7^ [33] |

Secretion rate in $\frac{pg}{mL {10}^{5} cells day}$

| *KE\|IL−*8 | EC secrete IL-8 | 234 [34] |
| --- | --- | --- |
| *^K^N_A_\|IL−*1*β*  *^K^N_A_\|TNF_α_* | AN secrete *IL −* 1*β*  AN secrete *TNF_α_* | 225 [35]  250 [38] |
| *^K^M*_1_*\|IL−*6  *^K^M*_1_*\| TNF_α_* | M1 secrete IL-6  M1 secrete *TNF_α_* | 250 [36]  70[36] |
| *^K^M*_2_*\|IL−*10  *^K^M*_2_*\|TGF_β1_* | M2 secrete IL-10  Rate of production of *TGF_β1_* by M2 | 45 [37]  280 [40] |
|  |  |  |
| *^K^N_DN_ \|IL−*8 | Rate of production of IL-8 by NN | 1*.*46 [39] |

*Decay rate(DR) per hour*

| *µ_IL−_*_8_ | DR of IL-8 | 0*.2* [41] |
| --- | --- | --- |
| *µ_IL−_*_1_*_β_* | DR of *IL −* 1*β* | 0*.*6 [42] |
| *µ_IL−_*_6_ | DR of IL-6 | 0*.*5 [43] |
| *µ_IL−_*_10_ | DR of IL-10 | 0*.*5 [44] |
| *µ_TNFα_* | DR of *TNF_α_* | 0*.1125*[45] |
| *µ_TGFβ1_* | DR of *TGF_β1_* | 0*.02* [46] |

*Endocytosis rate(ER) in* $\frac{pg}{ml {10}^{5} cells day}$

| *^θ^N_A_\|IL−*8 | ER of IL-8 by AN | 3*.*024 [39] |
| --- | --- | --- |

**Table S2**: Initial Description of Different Cell Types. (-) means that this value is unknown or dependent on the cell differentiation process. Life span in the simulation symbolizes the maximum amount given to the cell type to either die or differentiate, some values are simulation dependent.

| **Cell Type** | **Number** | **Initial Location** | **Movement** | **Mitosis** | **Life span** |
| --- | --- | --- | --- | --- | --- |
| Endothelial Cell | See Table 3 | Tissue | No | No | Immortal |
| Resting Neutrophil | 1000  100 | Blood Tissue | Yes  Yes | No  No | 120h [47]  120h [47] |
| Monocyte | 1000 | Blood | Yes | No | 24h [48] |
| Fibroblast | 100 | Tissue | Yes | No | Immortal |
| Macrophages type I | - | Tissue | Yes | No | Immortal |
| Macrophages type II | - | Tissue | Yes | No | Variable in time, immortal after 96h |
| Myofibroblast | 25 | Tissue | Yes | No | Immortal |

**Table S3**: (a) Coefficients from the sigmoid function and (b) Cytokine concentration Transformations used. These transformations are useful to obtain calculations in the same order of magnitude.

| **Coefficient(s)** | **Value** | **Cytokine** | **Transformation** |
| --- | --- | --- | --- |
| *w*_1_*, w*_2_*, w*_3_*, w*_4_ | 0.25 | *IL −* 8 | *×* 10^9^ |
| *w*_5_*, w*_9_ | 0.5 | *IL −* 1*β* | *×* 10^9^ |
| *w*_6_ | 0.9 | *TNFα* | *×* 10^9^ |
| *w*_7_*, w*_8_ | 0.45 | *TGF_β1_* | *×* 10^10^ |
| *w*10 | 0.9 | *IL −* 6 | *×* 10^11^ |
| *w*11 | 0.1 | *IL −* 10 | *×* 10^12^ |

1. (b)

**Table S4**: Plugin modules used in the simulation.

| **Plugin Name** | **Function** |
| --- | --- |
| CellType | List all cell types with specific IDs and colors |
| CenterOfMass | Track center of mass of each cell |
| PixelTracker | Track pixels of each cell |
| Contact | Specify adhesion energies |
| DiffusionSolverFE | Specify diffusion field and PDE solvers |
| Chemotaxis | Specify chemotaxis properties of select cell types |
| Connectivity | Apply connectivity constraint to each cell |
